# Effect of Quorum Sensing by *Staphylococcus epidermidis* on the Attraction Response of Female Adult Yellow Fever Mosquitoes, *Aedes aegypti aegypti* (Linnaeus) (Diptera: Culicidae), to a Blood-Feeding Source

**DOI:** 10.1371/journal.pone.0143950

**Published:** 2015-12-16

**Authors:** Xinyang Zhang, Tawni L. Crippen, Craig J. Coates, Thomas K. Wood, Jeffery K. Tomberlin

**Affiliations:** 1 Department of Entomology, Texas A&M University, College Station, Texas, 77843, United States of America; 2 Department of Zoology, University of Cambridge, Cambridge, CB2 3EJ, United Kingdom; 3 Southern Plains Agricultural Research Center, Agricultural Research Service, USDA, College Station, Texas, 77843, United States of America; 4 Department of Chemical Engineering, Pennsylvania State University, State College, Pennsylvania, 16802, United States of America; University of California Davis, UNITED STATES

## Abstract

*Aedes aegypti*, the principal vector of yellow fever and dengue fever, is responsible for more than 30,000 deaths annually. Compounds such as carbon dioxide, amino acids, fatty acids and other volatile organic compounds (VOCs) have been widely studied for their role in attracting *Ae*. *aegypti* to hosts. Many VOCs from humans are produced by associated skin microbiota. *Staphyloccocus epidermidis*, although not the most abundant bacteria according to surveys of relative 16S ribosomal RNA abundance, commonly occurs on human skin. Bacteria demonstrate population level decision-making through quorum sensing. Many quorum sensing molecules, such as indole, volatilize and become part of the host odor plum. To date, no one has directly demonstrated the link between quorum sensing (i.e., decision-making) by bacteria associated with a host as a factor regulating arthropod vector attraction. This study examined this specific question with regards to *S*. *epidermidis* and *Ae*. *aegypti*. Pairwise tests were conducted to examine the response of female *Ae*. *aegypti* to combinations of tryptic soy broth (TSB) and *S*. *epidermidis* wildtype and *agr-* strains. The *agr* gene expresses an accessory gene regulator for quorum sensing; therefore, removing this gene inhibits quorum sensing of the bacteria. Differential attractiveness of mosquitoes to the wildtype and *agr-* strains was observed. Both wildtype and the *agr-* strain of *S*. *epidermidis* with TSB were marginally more attractive to *Ae*. *aegypti* than the TSB alone. Most interestingly, the blood-feeder treated with wildtype *S*. *epidermidis*/TSB attracted 74% of *Ae*. *aegypti* compared to the *agr-* strain of *S*. *epidermidis*/TSB (*P* ≤ 0.0001). This study is the first to suggest a role for interkingdom communication between host symbiotic bacteria and mosquitoes. This may have implications for mosquito decision-making with regards to host detection, location and acceptance. We speculate that mosquitoes “eavesdrop” on the chemical discussions occurring between host-associated microbes to determine suitability for blood feeding. We believe these data suggest that manipulating quorum sensing by bacteria could serve as a novel approach for reducing mosquito attraction to hosts, or possibly enhancing the trapping of adults at favored oviposition sites.

## Introduction

Mosquitoes are important vectors responsible for the transmission of viruses, bacteria, parasitic protozoans, and filarial, which cause diseases in humans; such as dengue fever, yellow fever, and malaria [1]. Among all vectors that transmit pathogens that cause disease, mosquitoes are widely regarded as the most dangerous to humans in terms of their efficiency as a vector, and resulting mortality, incapacitation, and economic losses. More than three billion people are threatened by pathogens transmitted by mosquitoes [2]. Pesticides and environmental management are still primarily used to eliminate mosquitoes, regardless of environmentally detrimental effects [3]. Genetic manipulation using molecular biology techniques and the development of new effective repellents have been increasingly touted as being important for the future control of mosquito-borne diseases [4].

In addition to heat and carbon dioxide cues, mosquitoes locate their hosts through olfaction systems sensing chemical cues emanating from humans [5]. As determined previously, human sweat is odorless unless incubated with bacteria [6]. It has also been shown that the bacteria on human skin play a significant role in the interactions between mosquitoes and their hosts, by producing odors that are attractive to mosquitoes [7]. Many of the volatiles emitted by the human body, to which mosquitoes respond, are produced by bacteria [8]. Many chemical compounds have been demonstrated to attract mosquitoes; such as estrogens, amino acids, fatty acids, aldehydes, carboxylic acids, alcohols, aliphatics/aromatics, amides, amines, esters, halides, heterocyclics, ketones, sulfides, and thioesters [9–11]. Other attraction cues include L-lactic acid, 1-octen-3-ol, acetone, and ammonia, which play important roles in host-seeking behavior over longer distances [9].

*Staphylococcus epidermidis* is a commensal bacteria associated with human skin that can attract mosquitoes [12]. *Anopheles gambiae* (Diptera: Culicidae), a species that vectors the malaria causative agent, were attracted more by blood agar plates incubated with *S*. *epidermidis* than by sterile blood agar plates [12]. Volatile organic compounds (VOCs) resulting from bacteria can be produced either by metabolism or by quorum sensing (QS), a cell-cell communication system in bacteria [13]. For example, putrescine [14] and indole [15, 16] are QS compounds produced by bacteria associated with humans that can volatilize and are known mosquito stimulants [17–20].

As a predominant bacterium associated with human skin [21], *S*. *epidermidis* contributes to the formation of volatile fatty acids [22], a VOC combination with a distinctive sweaty odor that is attractive to mosquitoes [10]. Currently, two QS systems of Gram-positive Staphylococci have been studied: the accessory gene regulator (*agr*) and *luxS* systems [23], which regulate a diverse array of physiological activities; including symbiosis, virulence, competence, conjugation, antibiotic production, motility, sporulation, and biofilm formation [13]. In QS, a variety of compounds are generated and released by the cells, many of which are known to volatilize.

Interspecies interactions regulated by QS compounds produced by microbes have been identified for a number of systems [24]. Such systems function in both inter-species and interkingdom communication. They have been identified in a wide variety of bacteria and also extend to relationships between bacteria and eukaryotes, and host–pathogen interactions in both clinical and agricultural settings [24]. Zoospores from the seaweed, *Enteromorpha*, have been shown to detect the presence of bacteria (*Vibrio anguillarum*) through recognition of their auto-inducer produced via their QS system which subsequently attaches to the bacterial biofilms and begins growth [25]. Conversely, regulation of bacterial QS systems may also be influenced by eukaryotes. For example host immune activation signals–interferon-γ–binds to an outer membrane protein in *Pseudomonas aeruginosa* which activates its QS dependent virulence determinant so that it enhances its virulence phenotype [26]. A previous similar study demonstrated that a mutant *rfaL* strain of *Proteus mirabilis*, which is unable to swarm, a QS-controlled phenotype, is less attractive to the blow fly, *Lucilia sericata* (Diptera: Calliphoridae) [27]. Furthermore, compared to the wildtype *P*. *mirabilis*, the *rfaL* strain had fewer eggs deposited on it by the flies. Such interactions are speculated to regulate arthropod attraction and colonization of ephemeral resources, such as vertebrate carrion, which is an important aspect of nutrient recycling within larger ecosystems [28–30].

The *agr* operon, which encodes a *S*. *epidermidis* QS system, was deleted to construct the mutant *S*. *epidermidis* strain (Tü3298) that cannot regularly perform QS [31]. The goal of this research was to determine if agr-based QS by *S*. *epidermidis* contributes to attracting *Ae*. *aegypti* to a blood meal.

## Results

### Mosquito Response to Blood-Feeders Treated Solely with TSB

**Fig 1:**
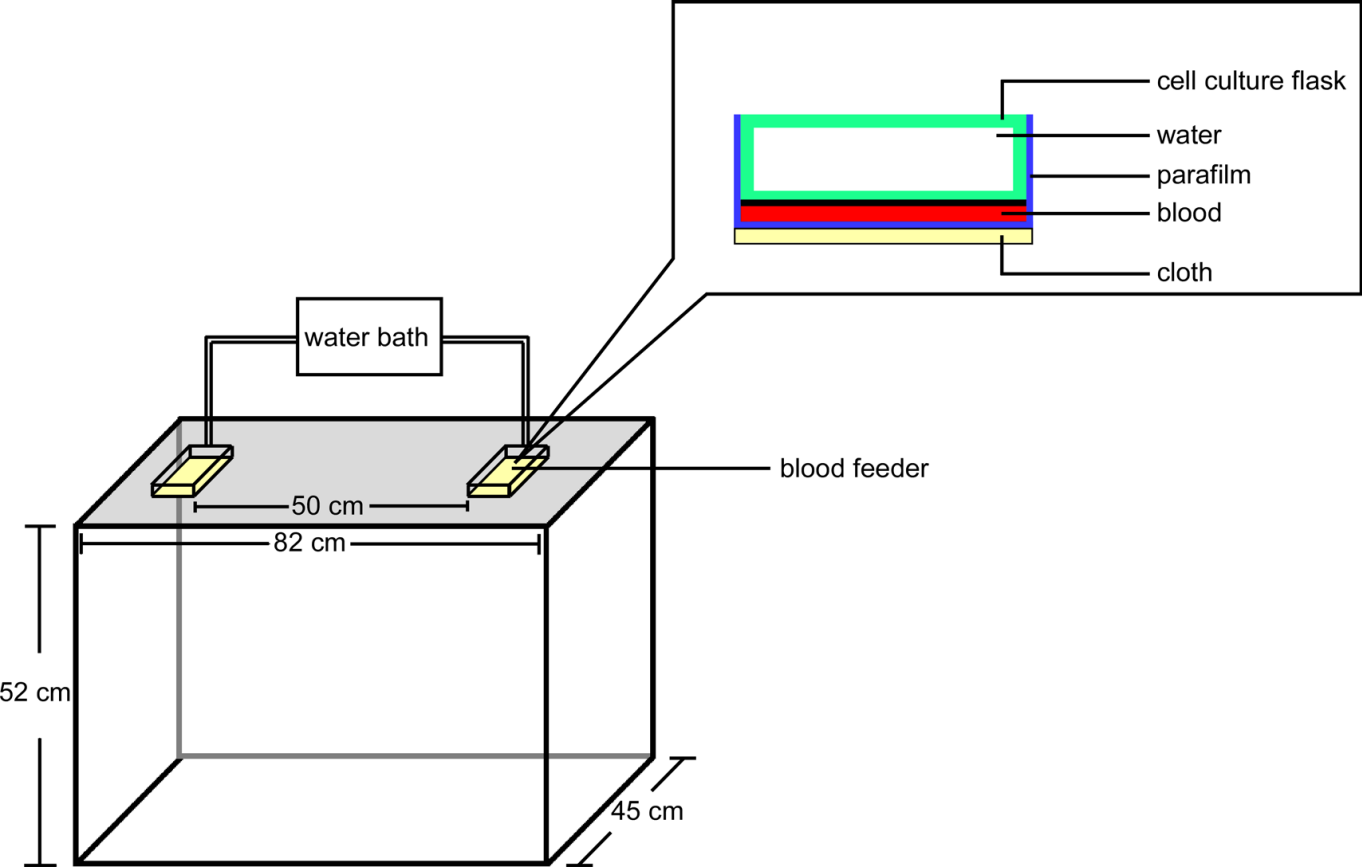
The blood-feeders used to conduct the behavioral assays with 5-8-d-old adult *Ae*. *aegypti* in the device.

The average percent response of adult female mosquitoes to the blood-feeders treated with TSB for each trial is presented in Table 1. Four trials of this experiment were conducted using a cage design (Fig 1). The blood-feeders located on the top left and right sides of the cage attracted a similar (approximately 50%) mean percent of mosquitoes per minute (Table 1).

**Table 1:** Repeated G test of goodness-of-fit test, percent response per trial, and mean percent ± SEM across trials of the response of 5-8-d old *Ae*. *aegypti* (N^1^ = 4; n^2^ = 50) adult female attraction to blood-feeders located on opposite sides of the top of a 82 cm x 45 cm x 52 cm Plexiglas cage during 15-minute experiments at 25°C and 80% RH and treated with solely TSB.

	G value	*P* value	Percent (total number^a^) mosquito response per trial	
			Left blood-feeder	Right blood-feeder
Trial 1	3.708	0.054	60.2% (53)	39.8% (35)
Trial 2	3.603	0.057	41.4% (51)	58.6% (72)
Trial 3	0.515	0.472	52.7% (38)	47.3% (32)
Trial 4	1.664	0.197	42.5% (31)	57.5% (42)
Total G	9.49	**0.049**		
Pooled G	0.018	0.671		
Heterogeneity G	9.309	**0.025**			
Mean ± SEM			49.2% ± 4.5%	50.8% ± 4.5%	

^1^number of trials conducted

^2^number of mosquitoes used in a trial

^a^Total number of mosquitoes to respond

**Fig 2:**
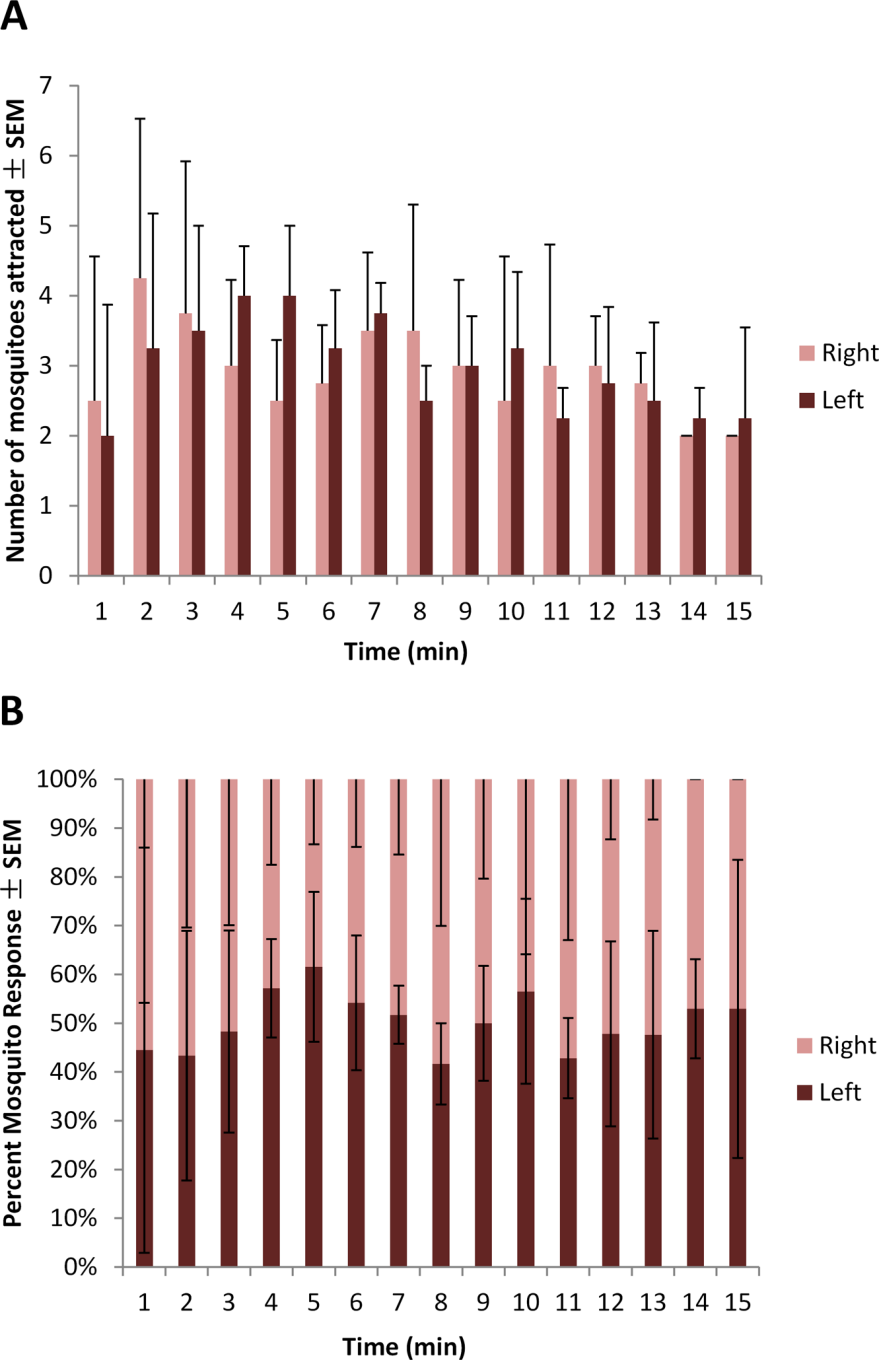
Mean number (A) and (B) percent of 5-8-d-old adult female *Ae*. *aegypti* mosquitos attracted per minute ± SEM to blood-feeders treated with TSB located on the right and left sides of the top of a 82 cm x 45 cm x 52 cm Plexiglas cage during 15-min trials (N^1^ = 4; n^2^ = 50) at approximately 25°C and 80% RH. ^1^number of trials conducted; ^2^number of mosquitoes used in a trial.

Statistical analysis indicates a significant difference between trials (G_H_ = 9.309, df = 3, *P* = 0.025) (Table 1). However, no trial resulted in a significant difference in attraction of *Ae*. *aegypti* between blood-feeders on the left or right side of the cage, indicating no positional bias. Treatments in the following experiments were still rotated across trials to avoid potential bias. Average number of adult female mosquitoes to respond to the blood-feeders treated solely with TSB per minute for 15 min is shown in Fig 2A. Average percent of adult female mosquitoes to respond to the blood-feeders treated solely with TSB per minute for 15 min is shown in Fig 2B.

### Mosquito Response to Blood-Feeders Treated with Wildtype *S*. *epidermidis*/TSB or Solely TSB

**Fig 3:**
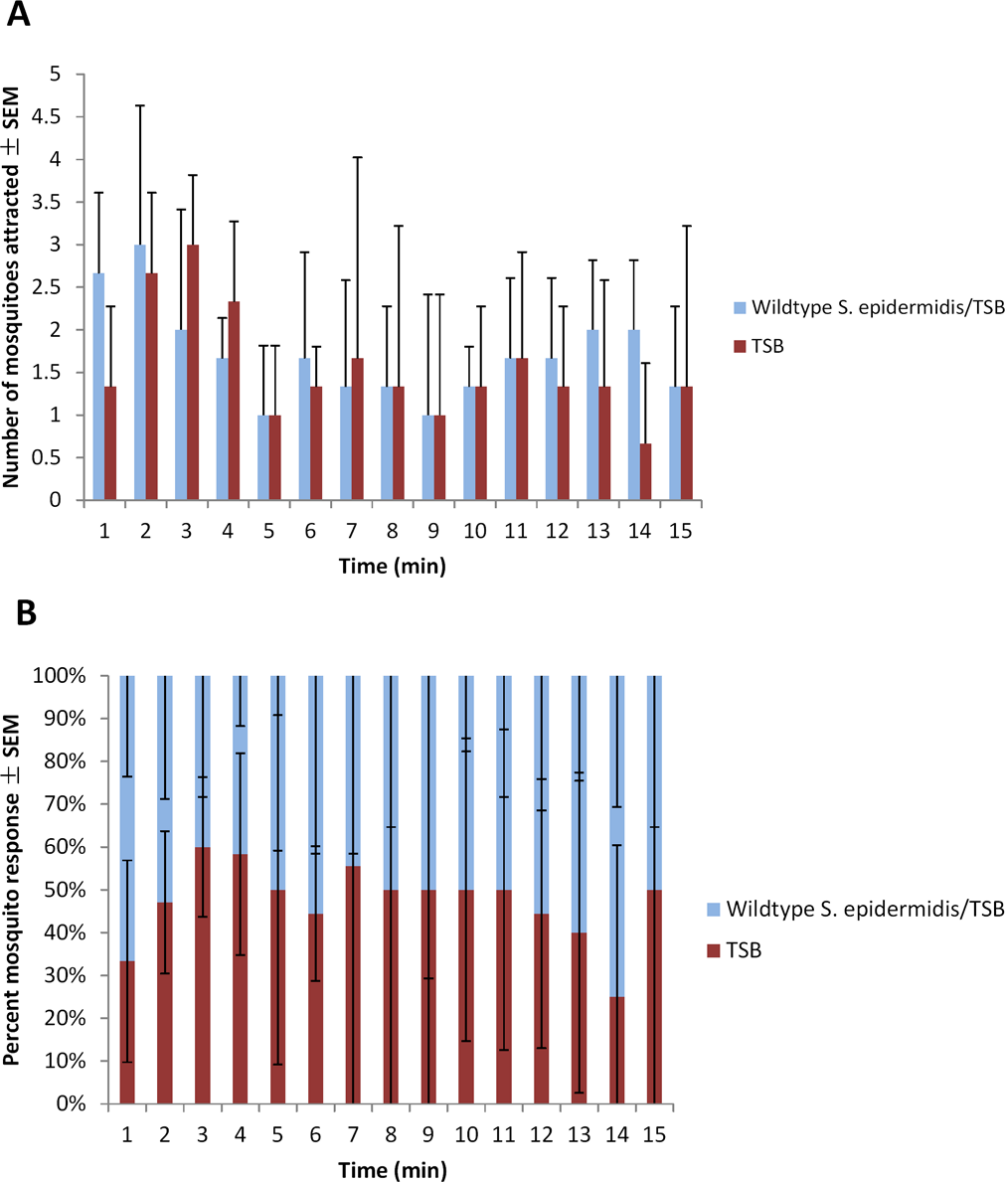
Mean number (A) and (B) percent of 5-8-d-old adult female *Ae*. *aegypti* mosquito attracted per minute ± SEM to blood-feeders treated with TSB or wildtype *S*. *epidermidis*/TSB located on the right and left sides of the top of a 82 cm x 45 cm x 52 cm Plexiglas cage during 15-min trials (N^1^ = 3; n^2^ = 50) at approximately 25°C and 80% RH. ^1^number of trials conducted; ^2^number of mosquitoes used in a trial.

Average percent response of adult female mosquitoes to the blood-feeders treated with wildtype *S*. *epidermidis/*TSB or solely TSB for each trial is presented in Table 2. Three trials of this experiment were conducted. The blood-feeder treated with wildtype *S*. *epidermidis*/TSB attracted, in general, 57% of the mosquitoes to respond (Fig 3B).

**Table 2:** Repeated G test of goodness-of-fit test, percent response per trial, and mean percent ± SEM across trials of 5-8-d old *Ae*. *aegypti* (N^1^ = 3; n^2^ = 50) adult female attraction to blood-feeders located on opposite sides of the top of a 82 cm x 45 cm x 52 cm Plexiglas cage during 15-min experiments at 25°C and 80% RH and treated with wildtype *S*. *epidermidis*/TSB or solely TSB.

	G value	*P* value	Percent (total number^a^) mosquito response per trial	
			Blood-feeder treated with wildtype *S*. *epidermidis*/TSB	Blood-feeder treated solely with TSB
Trial 1	0.297	0.586	46.3% (25)	53.7% (29)
Trial 2	0.243	0.622	46.7% (31)	53.3% (35)
Trial 3	8.826	**0.003**	77.8% (21)	22.2% (6)
Total G	0.333	0.564		
Pooled G	9.033	**0.011**		
Heterogeneity G	0.297	**0.025**		
Mean ± SEM			56.9% ± 9.0%	43.1% ± 9.0%

^1^number of trials conducted

^2^number of mosquitoes used in a trial

^a^Total number of mosquitoes to respond

Statistical analysis indicates a significant difference between trials (G_H_ = 0.297, df = 2, *P* = 0.025) (Table 2). One of the three trials demonstrated significant attraction to the blood-feeder treated with the wildtype *S*. *epidermidis*/TSB (Table 2). The remaining two trials were not significant for either treatment. The average number of adult female mosquitoes to respond to the blood-feeders treated with the wildtype *S*. *epidermidis* strain/TSB or solely with TSB per minute for 15 min is shown in Fig 3A. The average percent of adult female mosquitoes to respond to the blood-feeders treated with the wildtype *S*. *epidermidis* strain/TSB or solely with TSB per minute for 15 min is shown in Fig 3B.

### Mosquito Response to Blood-Feeders Treated with *agr- S*. *epidermidis*/TSB or Solely TSB

Average percent response of adult female mosquitoes to the blood-feeders treated with *agr-* strains of *S*. *epidermidis/*TSB or solely TSB for each trial is presented in Table 3. Four trials of this experiment were conducted. Similar to the previous results for the wildtype *S*. *epidermidis*, the blood-feeder treated with *agr-* strain *S*. *epidermidis*/TSB attracted, in general, 57% of the mosquitoes (Table 3).

**Table 3:** Repeated G test of goodness-of-fit test, percent response per trial, and mean percent ± SEM across trials of the response of 5-8-d old *Ae*. *aegypti* (N^1^ = 4; n^2^ = 50) adult female attraction to blood-feeders located on opposite sides of the top of a 82 cm x 45 cm x 52 cm Plexiglas cage during 15-min experiments at 25°C and 80% RH and treated with *agr-* mutant *S*. *epidermidis/*TSB or solely TSB.

	G value	*P* value	Percent (total number^a^) mosquito response per trial	
			Blood-feeder treated with *agr-* mutant *S*. *epidermidis*/TSB	Blood-feeder treated solely with TSB
Trial 1	1.526	0.217	55.9% (62)	44.1% (49)
Trial 2	6.059	**0.014**	76.2% (16)	23.8% (5)
Trial 3	0.257	0.612	54.3% (19)	45.7% (16)
Trial 4	0.611	0.434	43.9% (18)	56.1% (23)
Total G	8.453	0.076		
Pooled G	2.331	**< 0.001**		
Heterogeneity G	6.122	0.106		
Mean ± SEM			57.6% ± 6.7%	41.5% ± 6.7%

^1^number of trials conducted

^2^number of mosquitoes used in a trial

^a^Total number of mosquitoes to respond

**Fig 4:**
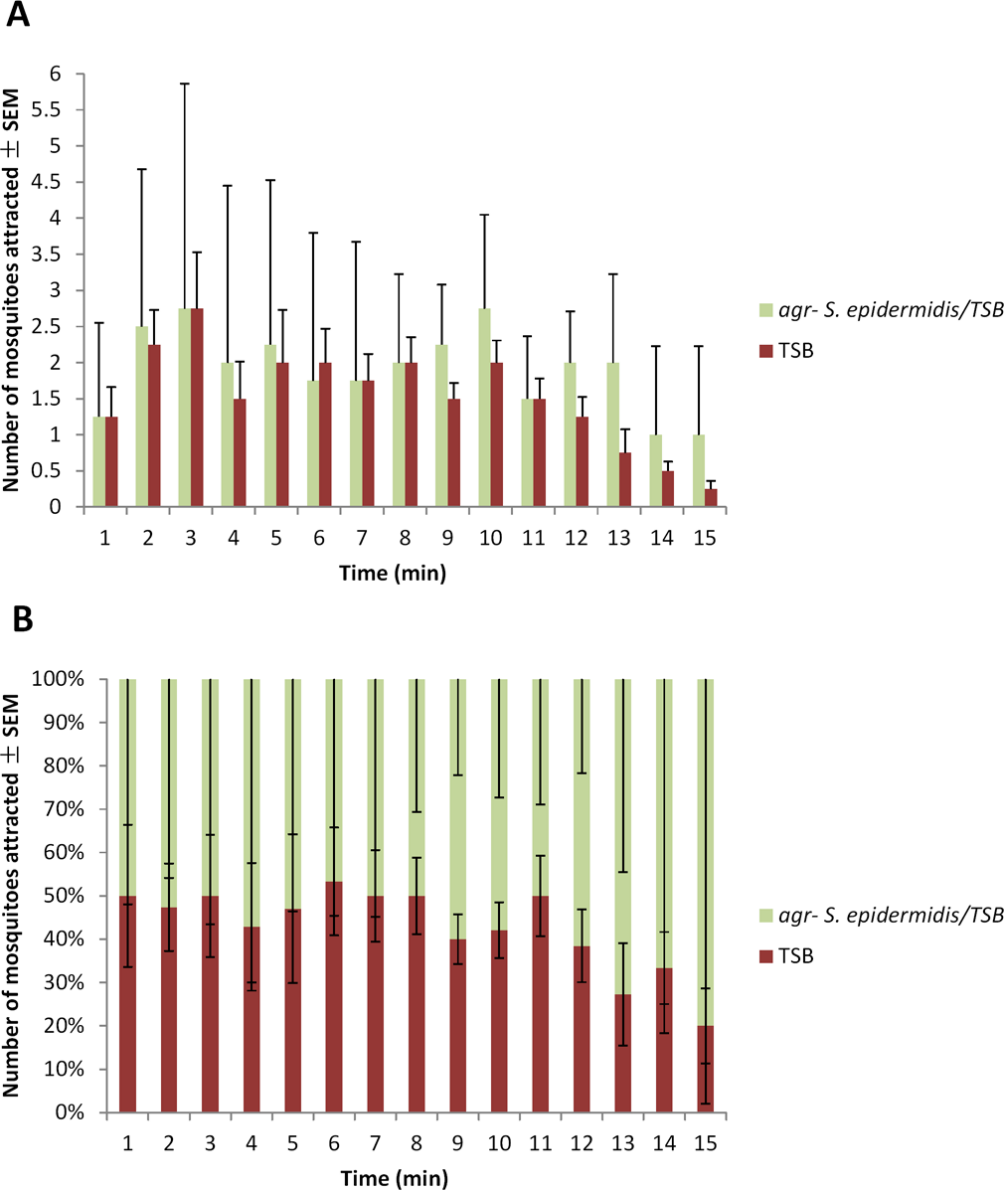
Mean number (A) and (B) percent of 5-8-d-old adult female *Ae*. *aegypti* mosquito attraction per minute ± SEM to blood-feeders treated with TSB or *agr- S*. *epidermidis*/TSB located on the right and left sides of the top of a 82 cm x 45 cm x 52 cm Plexiglas cage during 15-min trials (N^1^ = 4; n^2^ = 50) at approximately 25°C and 80% RH. ^1^number of trials conducted; ^2^number of mosquitoes used in a trial.

Statistical analysis did not indicate a significant difference between trials (G_H_ = 6.122, df = 3, *P* = 0.106) (Table 3). However, one of the four trials demonstrated significant mosquito attraction to the blood-feeder treated with the *agr-* strain of *S*. *epidermidis*/TSB rather than solely TSB (Table 3). The remaining three trials were not significant for either treatment (Table 3). The average number of adult female mosquitoes to respond to the blood-feeders treated the *agr- S*. *epidermidis* strain/TSB or solely with TSB per minute for 15 min is shown in Fig 4A. The average percent of adult female mosquitoes to respond to the blood-feeders treated with *agr- S*. *epidermidis*/TSB or solely TSB per minute for 15 min is shown in Fig 4B.

### Mosquito Response to Blood-Feeders Treated with Wildtype/TSB or *agr- S*. *epidermidis*/TSB

Average percent response of adult female mosquitoes to the blood-feeders treated with wildtype *S*. *epidermidis*/TSB or *agr- S*. *epidermidis*/TSB for each trial is presented in Table 4. Four trials of this experiment were conducted. The blood-feeder treated with wildtype *S*. *epidermidis*/TSB attracted 72% of the *Ae*. *aegypti* over the blood-feeder treated with the *agr-* strain of *S*. *epidermidis*/TSB (Table 4).

**Table 4:** Repeated G test of goodness-of-fit test, percent response per trial, and mean percent ± SEM across trials of the response of 5-8-d old *Ae*. *aegypti* (N^1^ = 4; n^2^ = 50) adult female attraction to blood-feeders located on opposite sides of the top of a 82 cm x 45 cm x 52 cm Plexiglas cage during 15-min experiments at 25°C and 80% RH and treated with wildtype *S*. *epidermidis*/TSB or *agr-* mutant *S*. *epidermidis*/TSB.

	G value	*P* value	Percent (total number^a^) mosquito response per trial	
			Blood-feeder treated with wildtype *S*. *epidermidis*/TSB	Blood-feeder treated with *agr-* mutant *S*. *epidermidis*/TSB
Trial 1	82.658	**<0.001**	72.2% (293)	27.8% (113)
Trial 2	147.364	**<0.001**	98.4% (119)	1.6% (2)
Trial 3	1.537	0.215	56.9% (45)	43.1% (34)
Trial 4	7.665	**0.005**	69.3% (70)	30.7% (41)
Total G	239.214	**< 0.001**		
Pooled G	164.813	**< 0.001**		
Heterogeneity G	74.401	**< 0.001**		
Mean ± SEM			74.2% ± 7.6%	25.8% ± 7.6%

^1^number of trials conducted

^2^number of mosquitoes used in a trial

^a^Total number of mosquitoes to respond

**Fig 5:**
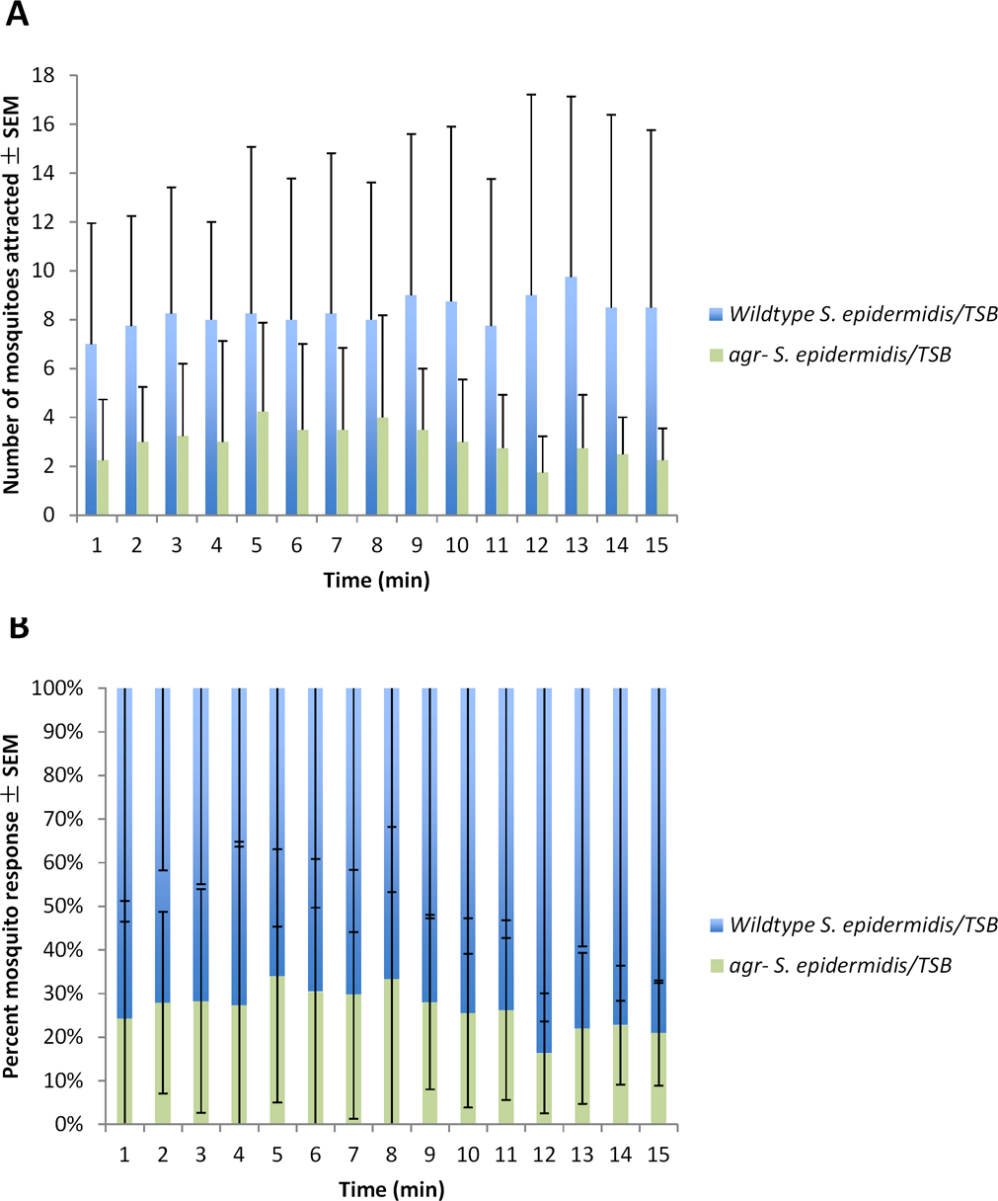
Mean number (A) and (B) percent of 5-8-d-old adult female *Ae*. *aegypti* mosquito attracted ± SEM to blood-feeders treated with wildtype or *agr- S*. *epidermidis*/TSB located on the right and left sides of the top of a 82 cm x 45 cm x 52 cm Plexiglas cage during 15-min trials (N^1^ = 4; n^2^ = 50) at approximately 25°C and 80% RH. ^1^number of trials conducted; ^2^number of mosquitoes used in a trial.

Statistical analysis did not indicate a significant difference between trials (G_H_ = 74.401, df = 3, *P* < 0.001) (Table 4). Three of the four trials indicated significant attraction to the wildtype *S*. *epidermidis*/TSB (Table 4). The remaining trial was not significant for either treatment. The average number of adult female mosquitoes to respond to the blood-feeders treated with wildtype *S*. *epidermidis*/TSB or *agr-* strains of *S*. *epidermidis*/TSB per minute for 15 min is shown in Fig 5A. The average percent of adult female mosquitoes to respond to the blood-feeders treated with wildtype *S*. *epidermidis*/TSB or *agr-* strains of *S*. *epidermidis*/TSB per minute for 15 min is shown in Fig 5B.

## Discussion

Bacteria living inside, as well as on a human body outnumber its cells by an estimated 10-fold [32]. The interactions, which include communication, between a human host and associated bacteria, as well as those interactions between the microbes themselves can be of vital importance for causing disease [33] through changes in host physiology and host signaling pathways. Furthermore, as suggested by our work, these interactions, especially between bacteria, could serve as a mechanism allowing for detection and determination of suitable hosts by vectors, such as the yellow fever mosquito, *Ae*. *aegypti*.

This “interkingdom cross-talk” between bacteria and eukaryotic organisms has gained increased recognition in recent years [34]. Some hosts, like *Vibrio fischeri*, are able to respond to bacterial QS and conversely produce molecules that are sensed directly by bacteria [35], resulting in an anti-predatory behavior called counter-illumination. This molecular communication can help to maintain a relatively stable mutualistic symbiotic association between the host (*Euprymna scolopes*) and the bacterial symbiont (*Vibrio fischeri*). *Salmonella typhimurium*, a bacterial pathogen, uses the QS regulator QseC and QseE to detect norepinephrine–a hormone from vertebrate hosts such as mouse [36]. This ability to assess the host environment through interkingdom cross-talk allows the pathogen to successfully colonize a host by avoiding host defenses, while outcompeting indigenous microbiota [37]. The marine macro-alga, *Delisea pulchra*, can block bacterial QS by producing halogenated furanones that act as signal mimics [38]. A recent study has also shown that the gut bacterium, *Acetobacter pomorum*, of *Drosophila* (Diptera: Drosophilidae) is able to promote host growth rates and reduce sugar and lipid levels according to the activity of its pyrroloquinoline quinone-dependent alcohol dehydrogenase (PQQ-ADH) [37]. This interaction maintains the gut microbe mutualism and is involved in the energy balance and metabolic homeostasis of host animals. Increasing evidence has been provided on connections between the gut microbiota and animal behavior [39]. For example, depression-like behaviors are reduced in bacteria-free mice, with defects in the brain regions that control anxiety [40], when they are fed probiotic bacteria [41, 42]. Despite this progress, few research studies have focused on unraveling the interaction between mosquitoes and human-associated bacteria.

In the case of mosquitoes, VOCs produced by humans play a role in influencing their host-seeking behavior. For example, carbon dioxide (CO_2_) is a strong volatile cue for mosquito attraction over long distances [43]. Ammonia, lactic acid and carboxylic acids, all of which are produced by microorganisms, have also been demonstrated to be attractive to mosquitoes [12]. In addition to different VOCs, variation in VOC concentrations can also result in differential attractiveness to mosquitoes. For example, a higher concentration of butyl 2-methylbutanoate, butyl butyrate, and butyl acetate, at a 1:100 dilution in a basic blend caught significantly more mosquitoes than trapping devices baited with the basic blend alone [44].

Some studies have demonstrated that human-associated bacteria produce VOCs that influence the level of attraction of their host to mosquitoes. For example, *Corynebacterium minutissimum* and *Bacillus subtilis* are significantly attractive to *An*. *gambiae* [44]. In contrast, VOCs produced by *P*. *aeruginosa* were not attractive, and may even attenuate the effect of volatiles from other bacterial species [44]. The role of *Staphylococci* in converting branched-chain amino acids to short-chain volatile fatty acids often associated with sweat is also intriguing in terms of a potential effect on the host-seeking behavior of *An*. *gambiae* [45]. However, for many of these important host-locating VOCs, the mechanisms underlying their production are not known.

Quorum sensing by bacteria results in the production of VOCs, which then serve as a mechanism allowing for interkingdom interactions [34, 35]. *S*. *epidermidis* is a predominant bacterium associated with human skin [21]. It is known that aerobic bacteria can reach 10^7^ bacteria per cm^2^ of skin [46, 47], particularly in moist areas, such as the axilla and the web between the toes. More recent metagenomic analysis has shown that *Staphylococcus* and *Corynebacterium spp*. bacteria dominated areas such as armpits and soles of the feet [48]. In this study, the bacteria were investigated at a concentration of 10^7^ cfu as that approximates bacteria, including Staphyloccoci, in human nares and armpits [49]. We recorded similar results as Price et al. [50], which was discussed in the introduction. We determined *Ae*. *aegypti* attraction to blood-feeders treated with wildtype and *agr- S*. *epidermidis* strains, demonstrating a 2.6 times greater attraction to the wildtype strain (Table 4). Furthermore, experiments where we examined the response of mosquitoes to blood-feeders with bacteria or TSB were more attracted to the feeder that contained bacteria (both wildtype and *agr-* strains), compared to TSB alone.

Results were relatively consistent towards a given treatment; however, heterogeneity (*P* < 0.0001) among trials was determined for each set of experiments conducted (Tables 1–4) Although we standardized the research methods implemented, slight variability in abiotic factors like temperature, humidity and larval nutrition could have resulted in the heterogeneity observed. For example, temperature can affect the performance of mosquito olfactory system: the mean tonic spike activity of antennal receptor neurons of *Ae*. *aegypti* tended to be optimal at 26–28°C [51]. Humidity has also been observed to be a factor influencing the attractiveness of the mosquito. *Anopheles quadrimaculatus* Say (Diptera: Culicidae), as they were more attracted by a warmer with more humidified human emanations air stream [50]. Furthermore, larval nutrition has been demonstrated to play an important role in affecting adult female *Ae*. *aegypti* host-seeking behavior [52]. Future research should examine these factors in greater detail to determine their impact on bacteria-mosquito interactions and reduce the variability.

The statistical approach implemented for analyzing response data in these experiments indicate care should be taken by researchers when determining the methods they employ to analyze their behavior data. Some commonly employed analyses, such as chi-square, would overlook such variation as we observed in our experiments, which could be crucial for deciphering the factors (e.g., temperature, humidity, starvation period) influencing host-seeking behavior by mosquitoes. Less rigorous methods could also potentially mask (e.g., sample size for one trial saturates responses observed in other trials) true treatment effects leading to misleading interpretations of the behaviors observed.

Results from these experiments could be used to develop novel methods for shifting VOC profiles by manipulating the QS system of host associated bacteria, resulting in reduced mosquito attraction. In addition to host-seeking behavior, previous research also demonstrated that mosquitoes preferred to oviposit on substrates containing live microorganisms, rather than sterilized substrates [53], indicating that the location of oviposition sites by *An*. *gambiae* is highly associated with microorganisms. Ponnusamy et. al [54] reported that specific bacteria-associated carboxylic acids and methyl esters are possible oviposition stimulants for gravid *Ae*. *aegypti*. Based on these previous studies, QS systems of the bacteria associated with mosquito oviposition sites should also be explored for the potential development of new and effective methods for mosquito control.

## Materials and Methods

### Mosquito Colony

The *Aedes aegypti* colony originated from the Liverpool strain. *Aedes aegypti* eggs were hatched in a container with 1 L distilled water at 25°C, 70% RH and 12:12 L:D photoperiod. Larvae at approximately 2-d-old were separated into containers at a density of 100–200 larvae/L, for optimal larval growth [55]. Larvae were fed on a diet of 3 g fish food (TetraMin diet by Tetra) every other day. After pupation, the mosquitoes were transferred into small plastic cups (60 ml) with distilled water at a density of 50 mosquitoes/cup. Plastic cups containing pupae were placed inside a metal cage with cotton sleeves on one side. Adults were collected every day after emergence using an aspirator, and placed in a paper cup (1.5 L) with mesh top to allow mating. Mosquitoes were provided a 5% sucrose solution with damp cotton wool ad libitum before use. Adult mosquito colonies were maintained under conditions of 25°C, 80% RH and 12:12 L:D photoperiod. Females, starved for 24 h, at 5-8-d-old [56] were used in the attraction assay.

### Bacterial Culture

The *agr* deletion mutant *S*. *epidermidis* strain (*S*. *epidermidis* TüF38) was used in this experiment because of the deletion of QS agr gene. Both the mutant (*agr* ^*-*^) and wildtype *S*. *epidermidis* strains were grown in mannitol salt agar (MSA), as MSA contains a high concentration of NaCl (approximately 7.5%-10%), it is inhibitory to most other bacteria, and selective for gram-positive bacterium *Staphylococci* (Mannitol salt agar 7143, Neogen Corp. 2008). The bacteria were diluted to a concentration of 10^7^ cfu in tryptic soy broth (TSB), for use in the experiments.

### Blood-Feeder Design

Blood-feeders were designed based on the design of a current PhD student (personal communication Luciano Cosme). The blood-feeders were made by 25 cm^2^ cell culture flask (Corning Incorporated NY) (Fig 1). Parafilm were used to cover the surface of the blood-feeder (Fig 1), so that there was a gap space between the parafilm and the surface of blood-feeder. A piece of 100% cotton cloth was cut into 4.5 cm x 9.5 cm pieces and autoclaved. Then the sterile cotton cloth was soaked with 500 μl, 10^7^ cfu of *S*. *epidermidis* wildtype or *agr-* mutant in TSB that are in exponential growth phase and attached covering the parafilm with a rubber band.

### Experiment Design

A clear Plexiglas cage (Fig 1) containing 50 5-8-d old female mosquitoes that had not received a previous blood meal was put in the incubator room under conditions previously described. Mosquitoes were placed in the cage 15 min prior to each trial to allow acclimation to the environment. In each trial, two blood-feeders were placed parallel to one another on opposite ends of the top of the cage (Fig 1). The two blood-feeders were connected by tubes to a water bath at 37°C. Each blood-feeder was either covered with cloth (Kaufman) soaked with 0.5 ml of 10^7^ colony forming units (cfu)/mL of the *S*. *epidermidis* wildtype strain, mutant (*agr- S*. *epidermidis* TüF38) strain or 0.5 ml TSB solution respectively. An aliquot of 1 ml of rabbit blood (HemoStat Laboratories) was added in the gap space between the blood-feeder and the parafilm. Initial experiments indicated there was no bias towards either side of the cage (see below); however, treatments were still rotated after each trial. Furthermore, the cage and feeders were cleaned using a vacuum and ethanol between trials.

The experiments were performed 3–4 h before dark (chamber at 12:12 L:D), based on *Ae*. *aegypti* females actively seeking hosts several hours before sunset [57, 58]. A camcorder (SONY HDR) was placed outside the cage and aimed to shoot up towards the underside of the blood-feeder. The mosquitoes were recorded for 15 min after introduction of the feeder for every trial. Using methods described previously [59–61], the number of mosquitoes feeding on either treatment was counted at each minute over the 15 min recording. Response data indicate the mosquito response per minute was consistent over the course of the experiment. Therefore, the total number of mosquitoes present on each feeder was determined by summing the total number of mosquitoes present at each one-minute observation period. Four trials of each experiment were conducted, with each trial representing a different mosquito generation. Experiments were conducted as outlined in Table 5.

**Table 5:** Treatments in experiments examining female adult 5-8-d old *Ae*. *aegypti* mosquito responses to blood-feeders with different microbial treatments in 82 cm x 45 cm x 52 cm Plexiglas cage during 15-minute trials in a laboratory at approximately 25°C and 80% RH.

Experiment	Treatment 1	Treatment 2	Purpose
1	TSB	TSB	Optimizing the conditions and determine if TSB attracts or repels *Ae*. *aegypti*
2	TSB	Wildtype *S*. *epidermidis*/TSB	Determine if wildtype *S*. *epidermidis* attracts or repels *Ae*. *aegypti*
3	TSB	*agr*- *S*. *epidermidis*/TSB	Determine if *agr*- *S*. *epidermidis* attracts or repels *Ae*. *aegypti*
4	Wildtype *S*. *epidermidis*/TSB	*agr*- *S*. *epidermidis*/TSB	Determine differential attractiveness of *Ae*. *aegypti* to wildtype *S*. *epidermidis* and *agr- S*. *epidermidis*

### Statistical Analysis

In order to determine if the mosquito response was significantly different between treatments and trials, the data were analyzed using the repeated G test of goodness-of-fit (*P* ≤ 0.05) as it allowed us to determine if variation from the expected proportion (50:50 in our case) and significant variation across trials [27], unlike chi-square which only considers differences from expected proportions.

## Supporting Information

S1 Dataset The number of mosquitoes attracted to different treatment of each replicate from the experiments. (XLSX)
